# Minimally invasive treatment of traumatic high rectovaginal fistulas

**DOI:** 10.1007/s00464-015-4192-z

**Published:** 2015-04-07

**Authors:** Denis Mukwege, Ntakwinja Mukanire, Jacques Himpens, Guy-Bernard Cadière

**Affiliations:** Gynaecology and General Surgery, Panzi General Referral Hospital, Bukavu, South Kivu Democratic Republic of the Congo; Gastrointestinal Surgery, Saint-Pierre University Hospital, 322, rue Haute, 1000 Brussels, Belgium

**Keywords:** Fistula, Rectovaginal fistula, High rectovaginal fistula, Laparoscopy, Obstetrical fistula

## Abstract

**Background:**

We propose a new minimally invasive technique by laparoscopic approach which minimizes parietal damage and allows precise location of the fistula, hence reduces blind dissection.

**Methods:**

Ten consecutive patients suffering from a HRVF benefited from the described technique. Location and time frame were east of the Democratic Republic of Congo and September 2012 through January 2014. By laparoscopy, dissection of the mesorectum in the “holy plane” is taken posteriorly as distally on the sacrum as possible. Dissection subsequently continues laterally beyond the fistula in an effort to maximally circumvene the fistulous area where no plane of cleavage can be found. If the cleavage plane beyond the fistula addresses a healthy rectum, a suture of vaginal and rectal defect is performed. If the cleavage plane beyond the fistula involves significant laceration of the rectum, while leaving at least 2 cm of healthy rectum above the sphincter, rectal resection and colorectal anastomosis are performed. If the rectal laceration involves the distal 2 cm but halts short of the sphincter (large fistula), the pull-through technique is performed.

**Results:**

Of ten participants, four had large HRVF and two presented significant fibrosis. Three underwent simple suture of rectal and vaginal defect, one rectal resection and six a “pull-through” technique. The median procedure time was 1h50 (1h00–3h30). There was no morbidity. None of the patients required protective ileostomy or colostomy. Nine patients were declared clinically cured with a median follow-up of 14.3 months (11–36). The Cleveland Clinic Incontinence Score was 20 in all patients before the treatment and was significantly (*p* = 0.004) reduced to 2.6 [0–20] after the treatment.

**Conclusions:**

This minimally invasive technique allowed us to treat HRVF, including complex ones in ten patients without significant morbidity. Clinical success with a median follow-up of 14.3 months was 90 %.

Literature data on the laparoscopic approach of high rectovaginal fistulae (HRVF) are scarce [1–6]. Treatment of HRVF is challenging because of the significant amount of inflammation and fibrosis around the diseased area, which obscures the interrectovaginal cleavage plane. We propose a new minimally invasive treatment consisting of a laparoscopic approach which minimizes parietal damage and allows precise location of the fistula, hence reduces blind dissection. The critical factor in obtaining precise location of the fistula is previous extensive posterior dissection of the rectum in the “holy plane” which facilitates thorough mobilization. Depending on the extent of rectal damage ascertained after dissection, treatment may consist of simple suturing of the rectal and vaginal defect, partial resection of the rectum or total rectal resection according to the “pull-through” technique.

## Materials and methods

This is a prospective study on consecutive patients in the east of the Democratic Republic of Congo suffering clinically from HRVF. The patients were consecutive and were enrolled between September 2012 and January 2014. This study was allowed by the ethical committee of the Catholic University of Bukavu. HRVF was defined as a communication between the mid-third of the rectum and the upper third of the vagina. History of the patients allowed to establish the cause of the condition (obstetrical, iatrogenous and/or rape) and to differentiate if fecal incontinence was secondary to sphincter loss or to a rectovaginal fistula. Physical examination was performed by a senior surgeon and allowed to evaluate the degree of inflammation and fibrosis of the vaginal mucosa as well as to determine the location and diameter of the fistula. Vaginal fibrosis was evaluated according to the classification of Judith W Goh et al. [7] (type I: no or mild fibrosis (around fistula/vagina), and/or vaginal length >6 cm, normal capacity; type II: moderate or severe fibrosis, and/or reduced vaginal length, poor capacity; type III: fibrosis due to a special circumstance, e.g., postirradiation, …). The anal sphincter tonus was evaluated by simple digital rectal examination. The diameter of the fistula was estimated by a tape measurer: A fistula was considered large when diameter was over 2.5 cm. Colonoscopy was not systematically performed.

Patients’ characteristics including social status, cause of the fistula and surgical and obstetrical history were recorded. Fecal incontinence graded according to the Cleveland Incontinence Score [8] was recorded before and after the procedure (Tables 1, 2).

**Table 1:**
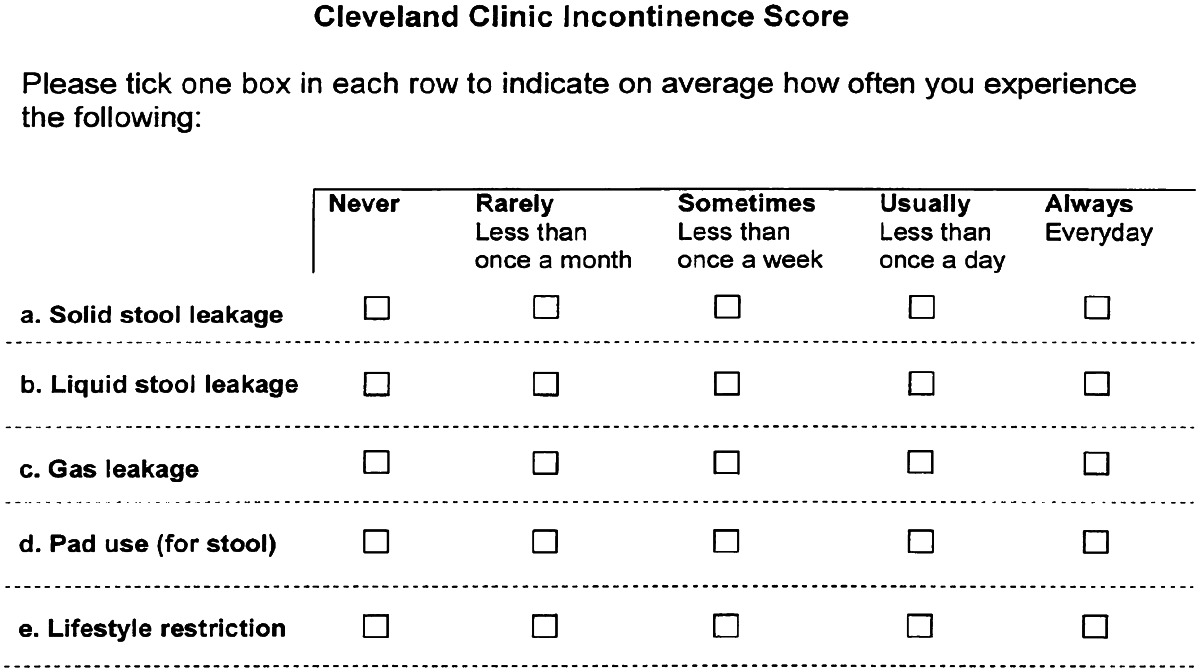
Cleveland Clinic Incontinence Score

**Table 2:**
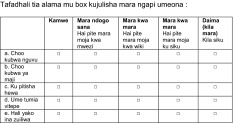
Translation of the Cleveland Incontinence Score from English to Kiswahili

Postoperatively, the patients were clinically reevaluated every 3 months. Fistula was considered healed when the loss of gas or stools per vaginam was no longer reported by the patient, when the vagina no longer displayed signs of inflammation or irritation and when transanal methylene blue irrigation performed with a vaginal tampon in place ruled out a fistula by lack of staining the tampon.

Cleveland Clinic Incontinence Scores were compared, in a paired way, using exact Wilcoxon signed-rank test.

The distributions of duration of hospital stay were compared according to two categorical variables (marital status and type of surgical intervention) using exact median two sample tests. A two-sided *p* value < 0.05 was considered significant. Because of the small size of the sample, the outcome data statistical analysis should be interpreted with care.

### Technique

**Fig. 1:**
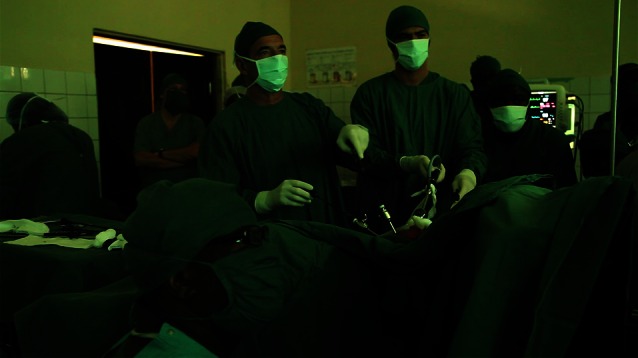
Disposition of the surgical team

**Fig. 2:**
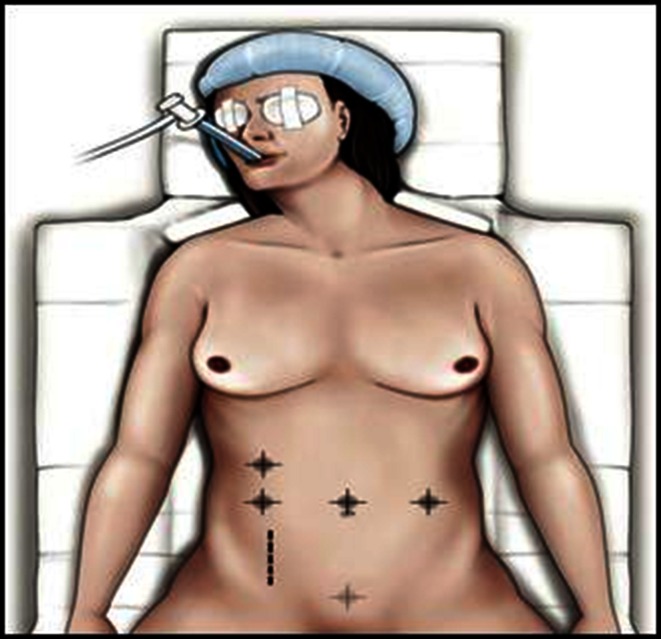
Disposition of the trocars

The patient is placed supine, legs apart. The technique is essentially a four-hand approach, with the gynecologist placed between the legs and the abdominal surgeon to the patient’s right (Fig. 1). Five trocars are used for the intervention: One 12-mm trocar is placed on the midline in a suprapubic position, one 10-mm trocar is placed at the umbilicus and three 5-mm trocars are placed in the right and left flank at the level of the umbilicus (Fig. 2). A 30° angled scope is used.

### Posterior mobilization of the rectum

The procedure is initiated by incising the peritoneum 1 cm ventral to the promontory. The assistant lifts the rectum cephalad.

**Fig. 3:**
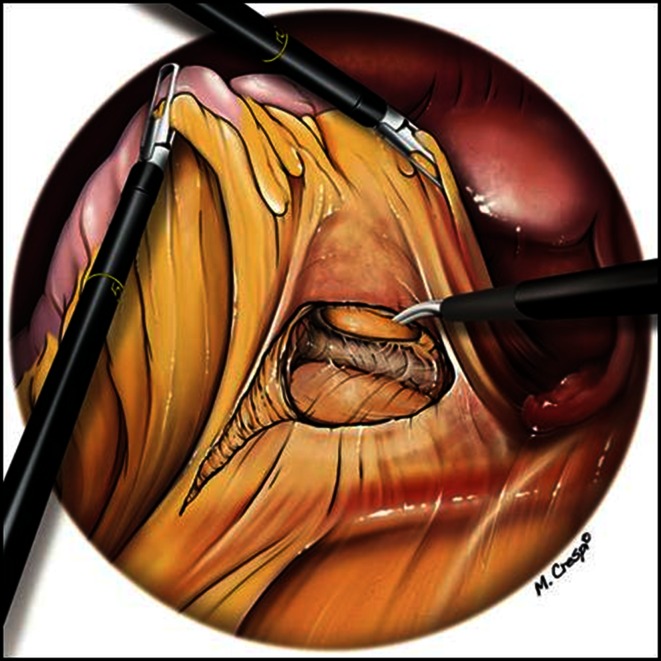
Opening of the holy plane

**Fig. 4:**
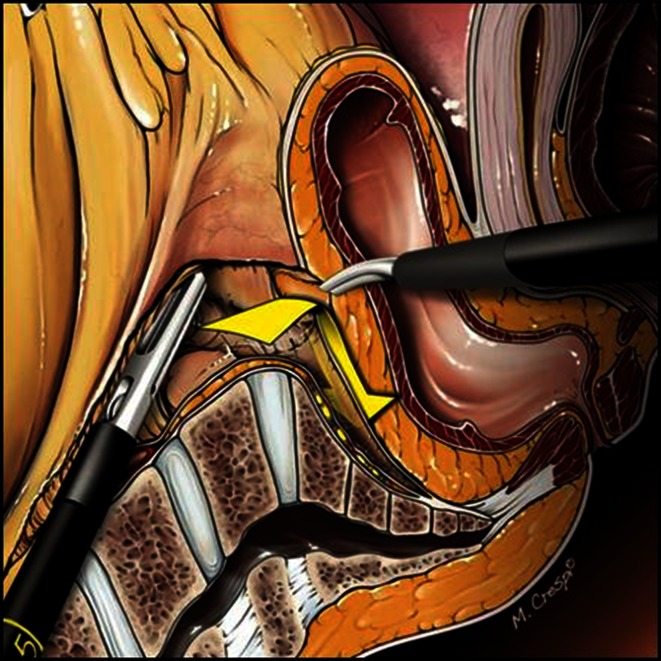
Opening of the holy plane

During posterior dissection of the rectum, one must resist the temptation to enter the straightforward plane indicated by the pneumodissection. This plane located deep to the presacral fascia is too posterior, and dissection in this plane may jeopardize the integrity of the presacral venous plexus and the hypogastric nerves. The correct plane can only be found by active dissection. A helpful landmark is the specific aspect of slightly different fat around the rectum, indicative of the right space located between the presacral fascia and the fascia propria of the rectum (Figs. 3, 4). This avascular plane was named the “holy plane” by Bill Heald. It is well described as it represents one of the essential steps in the total mesorectal resection.

Because the dissection is carried out anterior to the presacral fascia the sympathetic chain, the median sacral artery and the rectal plexus stay out of harm’s way.

**Fig. 5:**
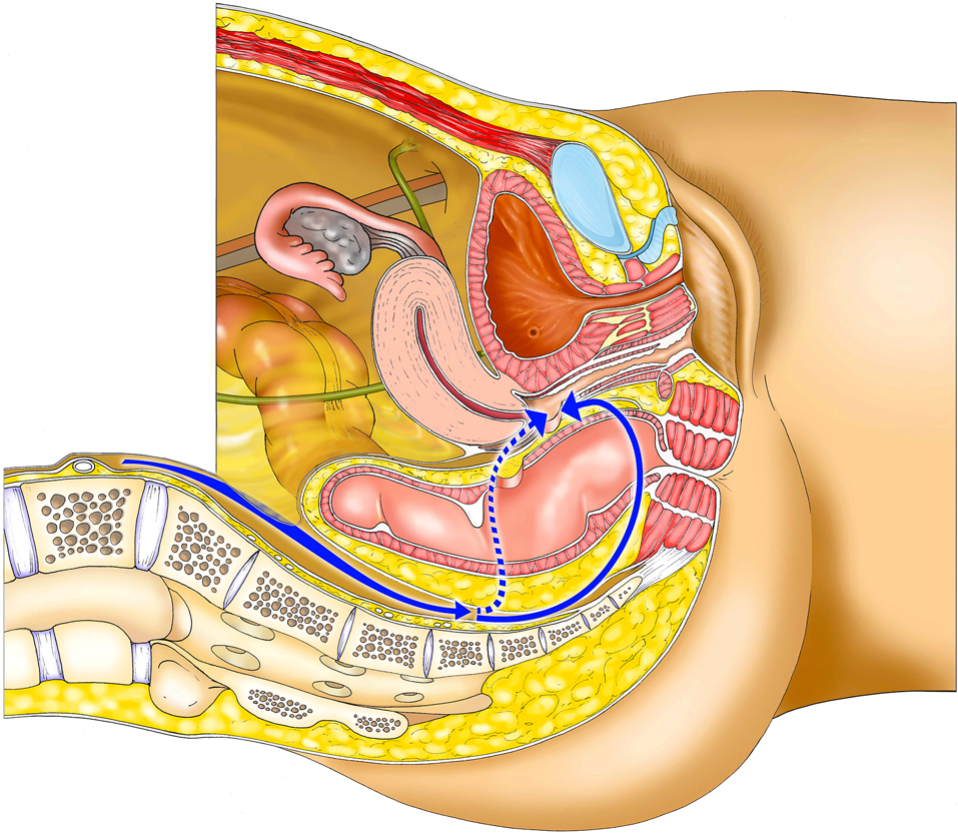
Step of dissection

**Fig. 6:**
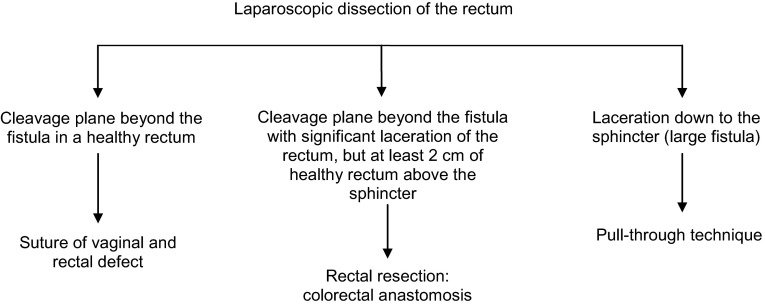
Operative strategy algorithm

Dissection is taken posteriorly as distally on the sacrum as possible and subsequently continued laterally beyond the fistula in an effort to maximally circumvene the fistulous area where no plane of cleavage can be found. Lateral dissection between the presacral fascia and the fascia propria is halted proximally to the rectal stalk. Hence, no resistance is felt during dissection from posterior to anterior. Subsequent anterior dissection is greatly facilitated by the previous posterior mobilization of the rectum and involves the dissection of the vaginal septum beyond the fistula (Fig. 5). Dissection of this latter structure is, however, not always feasible. When this step is deemed impossible, the surgical team elects the “pull-through” technique ab initio. Conversely, when anterior dissection beyond the fistula is estimated feasible and provided the rectum is not too severely damaged, the first option is selected, i.e., simple suture of the rectal and vaginal defect (Fig. 6).

### Rectal and vaginal suture

**Fig. 7:**
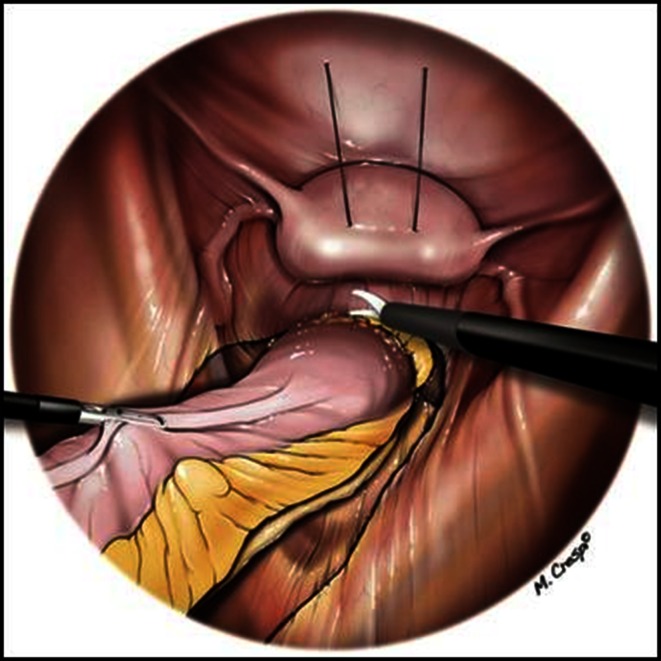
Dissection of the fistula

The dissection of the fistula itself is performed with the harmonic scissors and is oriented slightly anteriorly toward the vagina, in an effort to obtain as small a rectal defect as possible (Fig. 7).

**Fig. 8:**
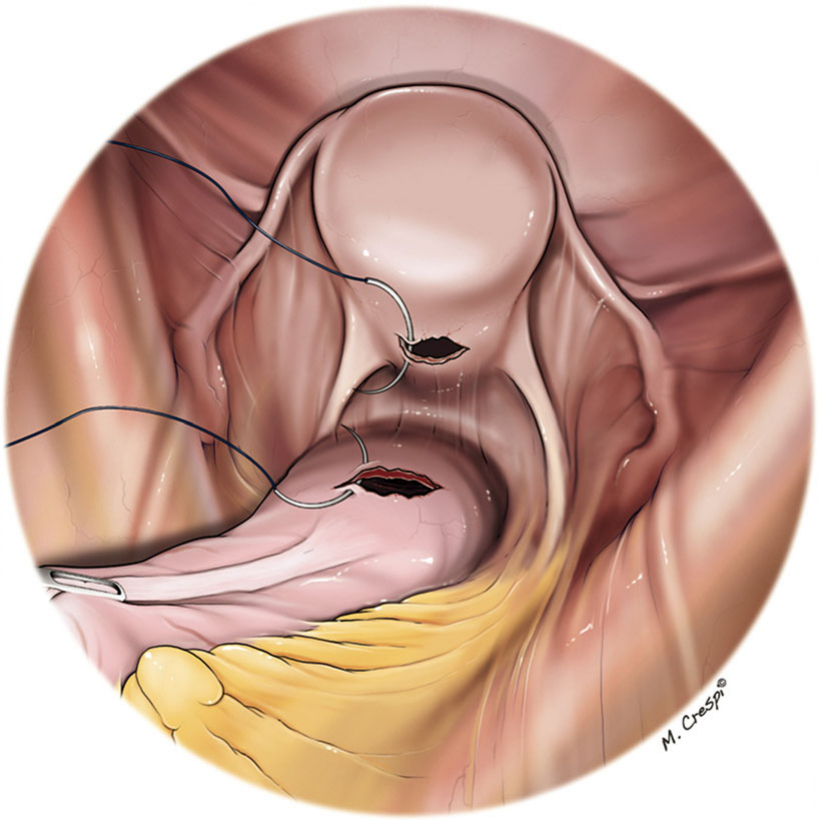
Vaginal and rectal suture

Loss of pneumoperitoneum is prevented by intravaginal placement of a surgical glove that is inflated. Vaginal suturing is carried out by separate “figure of 8” stitches of Vicryl 2/0 and is terminated as soon as the pneumoperitoneum is no longer escaping (Fig. 8).

**Fig. 9:**
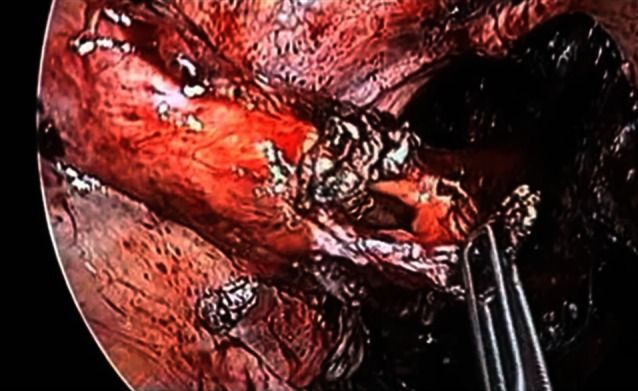
Suture of rectal defect

**Fig. 10:**
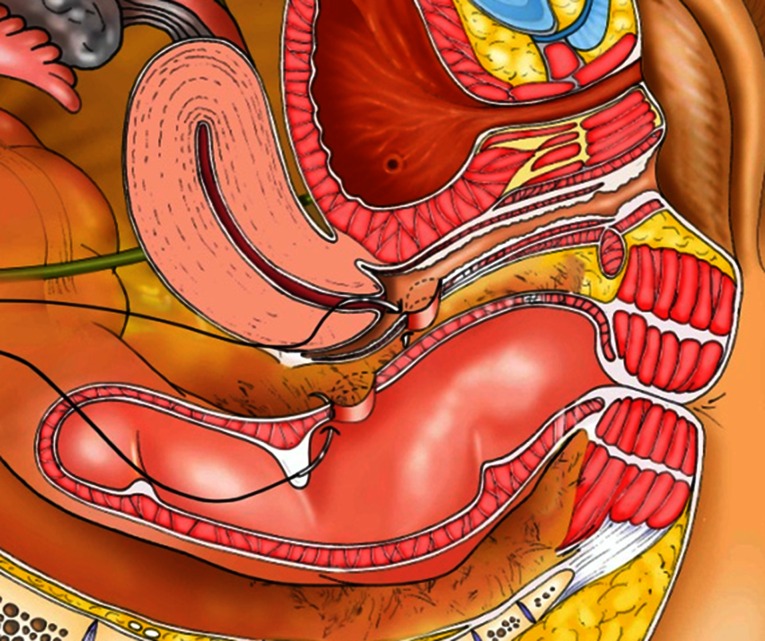
Position of the vaginal and rectal suture after extensive mobilization

The fibrosis around the fistula is trimmed as well as the edges of the rectal defect which is then closed by separate stitches of PDS 2/0. Thanks to the mobilization of the rectum, the vaginal and rectal defects are no longer placed at the same level (Figs. 9, 10). A greater omentum flap further protects the rectal suture.

### Rectal resection

When the surgeon manages to find a plane of dissection beyond the fistula and provided the rectum appears healthy from 2 to 3 cm proximal to the sphincter, rectal resection and colorectal reanastomosis may be attempted. This technique requires mobilization of the left colon. Toldt’s fascia is opened at the level of the angle of Treitz. The inferior mesenteric artery is dissected beyond its bifurcation into left colic and sigmoid artery. The latter is sectioned between two clips. The opening of Toldt’s fascia is continued until the presacral fascia (which lies in its continuity) is reached. The left paracolic gutter is dissected.

After full mobilization of the left mesocolon, a linear stapler is introduced in the 12-mm suprapubic trocar. The rectum is transected some 2–3 cm proximal to the sphincter using a 6-cm blue load. The damaged rectum and more proximal colon are extracted through a 3- to 4-cm Pfannenstiehl incision.

**Fig. 11:**
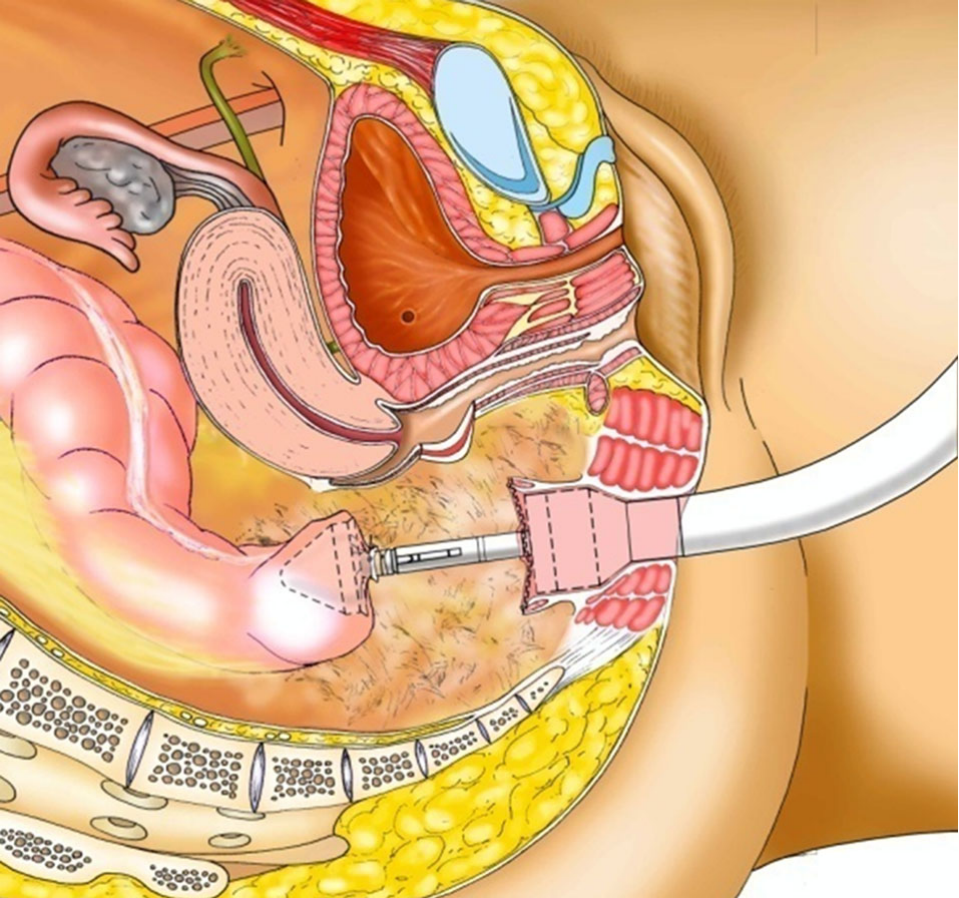
Mechanical circular stapler anastomosis

The lacerated rectum is resected, and the proximal end of the colon is reintegrated inside the peritoneal cavity. The anastomosis is performed transanally by circular stapler without routine diverting colostomy (Fig. 11).

### Technique of the “pull-through”

**Fig. 12:**
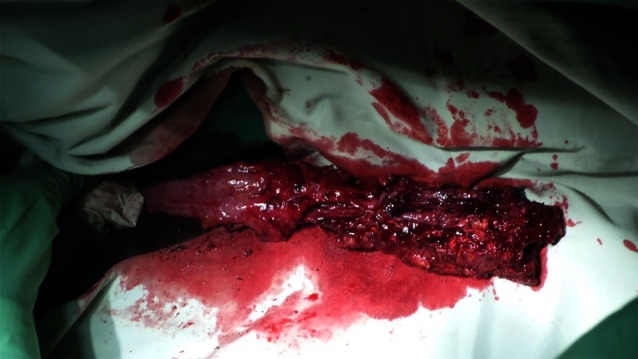
“Pull-through” technique

**Fig. 13:**
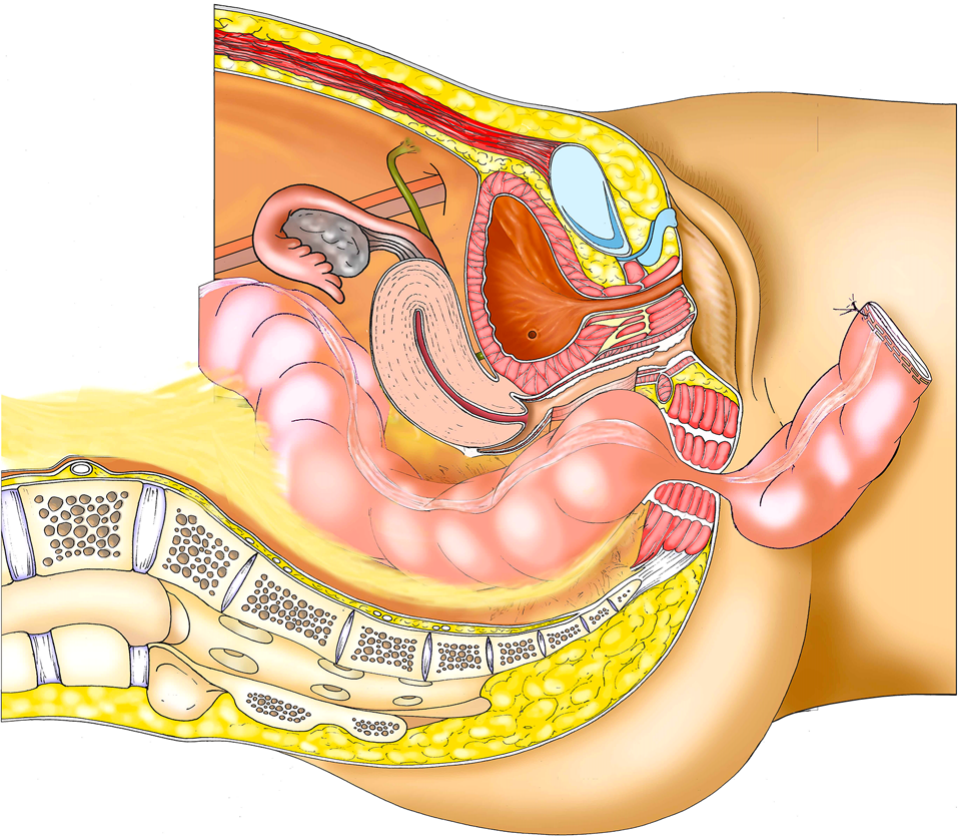
“Pull-through” technique

**Fig. 14:**
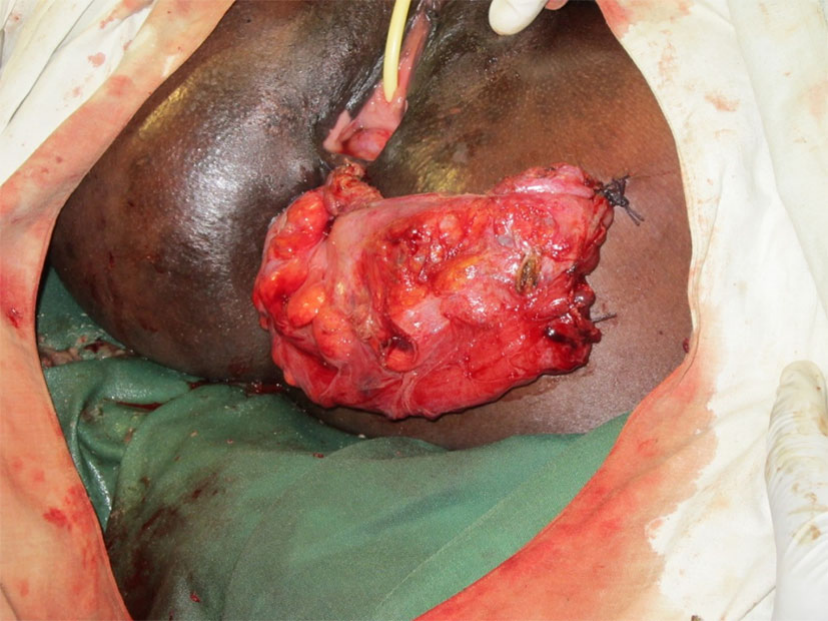
“Pull-through” technique

**Fig. 15:**
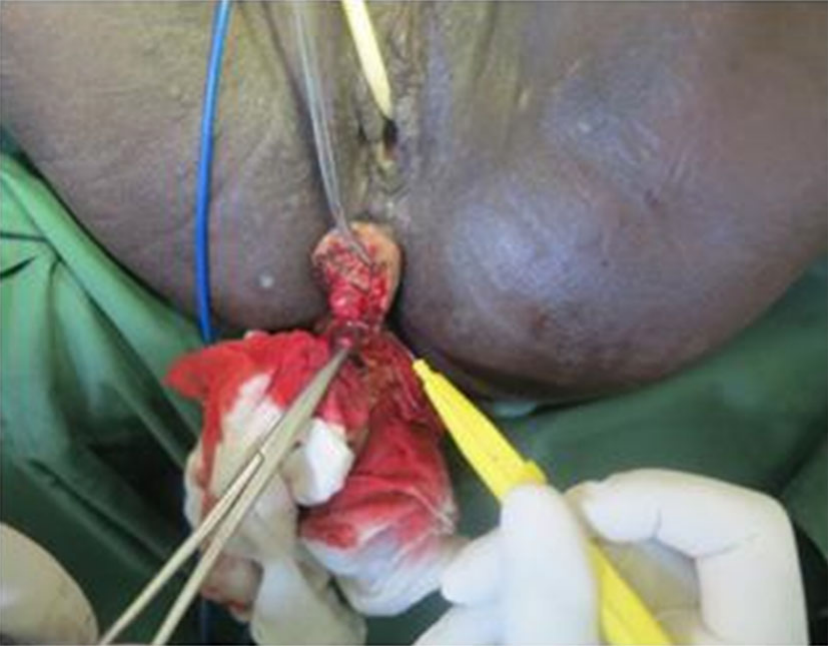
Recoupe of the stump at day 10

**Fig. 16:**
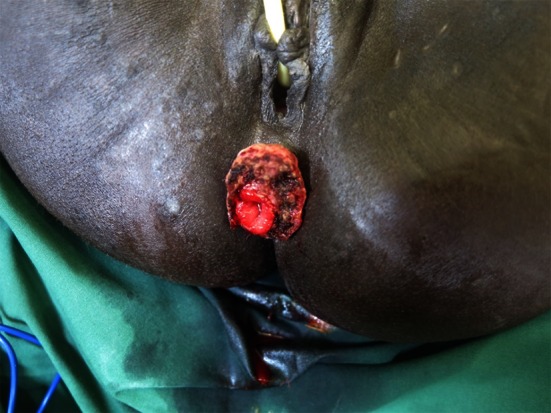
Final aspect of the recoupe of the stump

**Fig. 17:**
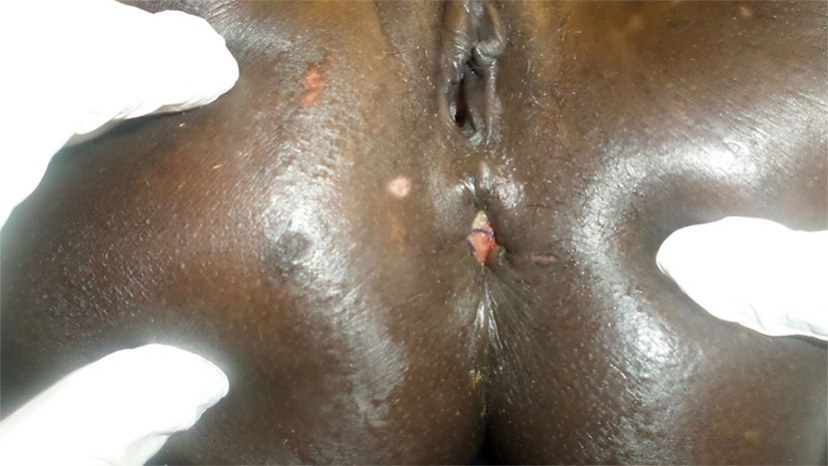
Intraperineal reduction in the trimmed stump

In cases where the rectal laceration stops just short of the anal sphincter (large fistula), the “pull-through” technique is preferred. With this technique, mobilization of the left colon must include full liberation of the splenic flexure. Laparoscopic dissection is subsequently continued to the levator, and the internal sphincter is identified. Rectal transection is performed a few millimeters proximal to the internal sphincter. The proximal rectum is snapped by an atraumatic grasper and pulled through the anus (Fig. 12). The lacerated rectal part is resected. The healthy part of the colon is pulled until some 10 cm protrude through the anus. This part of the colon is fixed to the inner thigh (Figs. 13, 14). Some 8 days later, the redundant colon is resected at 1 cm from the anus and fixed to it by 6 simple sutures (Figs. 15, 16, 17).

## Results

Between September 2012 and January 2014, 10 consecutive patients presented clinical evidence of a HRVF and benefited from the above-described surgical techniques (Table 3). Among the 10 patients, three underwent simple suture of rectal and vaginal defect, one rectal resection and six a “pull-through” technique.

**Table 3:** Characteristics, treatment and results of the 10 patients

Nbr	Length (cm)/Weight (kg)	Etiology–Obstetric–Iatrogenic–rape	Diagnosis	Surgery type	Hospital stay (days)	Physical exam	Cleveland Score/20	
							Before	After
1	152/45	Dystocic delivery/iatrogenic	*Ø* 3 cm Fibrosis type II FVV associated	“Pull-through”	20	Healed	20	0
2	151/47	Dystocic delivery	*Ø* 5 cm 1 cm from the AVFVV associated	“Pull-through”	21	Healed	20	6
3	125/40	Dystocic delivery	*Ø* 5 cm 4 cm from the AVFVV associated	“Pull-through”	26	Healed	20	0
4	148/48	Dystocic delivery	*Ø* 2 cm 4 cm of the anal verge	“Pull-through”	27	Healed	20	0
5	158/42	Dystocic delivery	*Ø* 2 cm 6 cm from the AV Fibrosis type II FVV Associated	“Pull-through”	25	Healed	20	0
6	141/45,5	Dystocic delivery	*Ø* 5 cm 2 cm from the AV FVV associated	“Pull-through”	36	Failed	20	20
7	149/48	Dystocic delivery	*Ø* < 1 cm 4 cm from the AV FVV associated	Rectal and vaginal suture	15	Healed	20	0
8	137/55,5	Dystocic delivery	*Ø* < 1 cm 7 cm from the AV	Rectal resection	21	Healed	20	0
9	148/43	Rape/vaginal perforation by wooden stick	*Ø* < 1 cm 8 cm from the AV FVV associated	Rectal and vaginal suture	10	Healed	20	0
10	147/48	Dystocic delivery	*Ø* < 1 cm FRV 7 cm from the AV FVV associated	Rectal and vaginal suture	15	Healed	20	0

*VVF* Vesicovaginal fistula, *AV* anal verge

The patients’ mean age was 26 (18–50) years. The mean length of the patients was 145.6 cm (125–152), and the mean weight was 46.2 kg (40–55). Seventy percent of the patients had been abandoned by their husband, relatives and fellow village inhabitants. Ninety percent of the patients were farmers. Seven patients had no children alive despite several pregnancies.

In terms of relevant surgical history, six patients (60 %) had undergone a cesarean section.

Eight patients suffered from an associated vesicovaginal fistula (VVF). Among these, seven had undergone several attempts at resolving the VVF without success.

The cause of the fistula was obstetric in eight patients and iatrogenic in one, and in one patient, the cause was an extremely violent rape with perforation of the vagina by a wooden pin. Another patient had suffered a rape followed by a pregnancy that resulted in a traumatic dystocic delivery.

Anal sphincter dysfunction was ruled out in all patients by history and clinical examination. All patients complained of loss of stools and gas per vaginam. Eighty percent of the patients presented concomitant urinary incontinence. The size of the rectovaginal fistula was over 2.5 cm in four patients (3–5–5–5 cm, respectively) and less than 2.5 cm in six (2–2–1–1–1–1 cm, respectively). Two patients presented type II vaginal fibrosis. The two patients who suffered from type II vaginal fibrosis were treated by “pull-through” technique, as were all the patients who presented large fistulas (over 2.5 cm).

The median operative time for the surgical procedures was 1h50 (1h00–3h30). Blood loss was evaluated at <25 cc in all cases, and none of the patients required red cell transfusion. In no patients, protective ileostomy or colostomy was performed.

Overall, the median hospital stay was 21 days (10–36): 25 days for abandoned women (*n* = 7) versus 15 days for married women (*n* = 3) (*p* = 0.07). There were no perioperative complications. The median hospital stay was 25.5 days for women who underwent the “pull-through” technique (*n* = 6) versus 15 days for the 3 women who had simple rectal and vaginal sutures (*p* = 0.17).

Nine of the 10 patients were declared clinically cured after a median follow-up of 14.3 months (11–36). One patient suffered a recurrent fistula after “pull-through” operation caused by retraction of the colonic stump at postoperative day 45.

The rating for fecal incontinence (Cleveland Clinic Incontinence Score) was documented both preoperatively and postoperatively in all 10 patients. Before the treatment, the score was 20 in all patients and was significantly (*p* = 0.004) reduced to 2.6 [0–20] after the treatment.

## Discussion

The incidence and etiology of RVF are highly correlated with the country’s degree of development [9, 10].

In the east of Congo, RVF are either obstetric–dystocic (caused by trauma during delivery), iatrogenic (suffered during a gynecological intervention) or purely traumatic (e.g., by extremely violent rape used as a weapon of war [11]). Additionally, rape may represent an indirect cause of HRVF whenever the victim is extremely young, becomes pregnant and ultimately gives birth while her pelvis is still underdeveloped (as in one of our patients) [12]. In our series, 80 % of the causes of RVF were dystocic–obstetric, one (10 %) was iatrogenic and one (10 %) was traumatic, namely an outrageously violent rape performed with a sharp wooden stick.

The high number of obstetrical accidents may find some explanation in the fact that our patients, even at mature age, were quite small and had a relatively narrow pelvis, which obviously may create problems during delivery. Nevertheless, the severity of obstetrical lesions suffered by our patients and the low number of children surviving despite several pregnancies demonstrate the difficulties in terms of obstetrical care in this region.

All fistulas were clinically obvious. The Cleveland Clinic Incontinence Score, aiming at giving a severity score to fecal incontinence, preoperatively was maximum in all patients and was confirmed at physical examination. Ancillary examinations to confirm the diagnosis were judged redundant. Consequently, colonoscopy was not systematically performed, moreover, because the main purpose of this endoscopy is to diagnose inflammatory bowel disease, a rare condition in this part of the world. Other diagnostic techniques such as CT scan and ultrasound were not available.

Traumatic high rectovaginal fistula is a devastating condition for the patient. The passage of stools and gas per vaginam is a tragic physical as well as psychosocial situation as demonstrated by the fact that 70 % of our patients had been socially abandoned.

Technically, HRVF constitute a substantial challenge to the surgeon especially because the surgical approach implies blind dissection, deep in the pelvis, in the space between vagina and rectum, in the fibrotic area around the fistula. In this regard, laparoscopy is an extremely useful tool because it allows enhanced visualization of the surgical planes of the deeper pelvis. At laparoscopy, dissection in this area is facilitated by a 30° angled scope.

The laparoscopic harmonic scissors are helpful in dissecting in the area of the fistula by keeping the surgical field dry. During “blind” dissection, it is wise to orient dissection toward the vagina because it digresses from the high-pressure zone of the fistula which is invariably situated in the rectum. Because of the high pressure at the rectum, stitch closure at that level must be completely airtight. Our dissection strategy allows us to minimize the size of the defect in the rectum after ablation of the fibrosis, hence makes it easier to close.

Previous dissection in the “holy plane” of the sacral hollow and freeing of the rectum from posterior to anterior allows adequate mobilization and correct identification of the rectum wall; hence, the blind part of the dissection is highly reduced. Dissection of the “holy plane” may be difficult in cases where necrosis is caused by compression as after difficult delivery, but this was not an issue in our experience of 10 cases. Extensive posterior dissection around the rectum may seem invasive, but in this particular area the plane is avascular and dissection does not create any functional sequelae. Rectal mobilization automatically places the vaginal and rectal suture at a distance from each other which theoretically reduces the risk of recurrence. Actually, we did not record any recurrence in our three rectal and vaginal sutures.

The choice between simple suture, rectal resection and “pull-through” (in increasing order of invasiveness) was made after full dissection of the fistula. Thorough dissection is required for adequate evaluation of the condition of the rectum, without jeopardizing the surgical options.

The option of simple suture is to be preferred whenever possible according to the findings of the full dissection because it eliminates the risk of anal incontinence and reduces the risk of recurrent fistula provided the level of the two fistula orifices can be altered.

In the rectal resection case, we did contemplate performing a diverting colostomy, but we decided against it because of the visibly excellent vascularity of the colonic stump.

Fistulas over 2.5 cm in diameter and accompanied by grade II fibrosis were systematically treated by the “pull-through” technique. This may be a safer option because extensive fibrosis of the vagina creates retraction of the vagina which impairs the prognosis of repair in that area [13]. Moreover, extensive vaginal fibrosis prevents adequate evaluation of the fistula size as well as of the distance to the anal verge.

The technique proposed here was fraught with low morbidity despite the complexity of the lesion in some patients. Conversely, hospital stay was quite long mostly because of social reasons. A majority (70 %) of the patients had been abandoned by their husband and family. It is a local hospital policy to take overall care of these patients. Thus, hospital stay was as long as 26 (21–36) days for the abandoned women versus 15 (10–20) days for the married women. In terms of surgical technique linked with hospital stay, the “pull-through” technique substantially lengthened the hospital stay to 25.8 days (20–36).

Postpartal anal sphincter lesions typically accompany rectovaginal fistulas and may explain the frequently documented persistence of anal incontinence after isolated repair of a postobstetrical rectovaginal fistula. Conversely, in our patient group discussed here, none suffered anal incontinence either before or after the surgical procedure. The absence of anal incontinence included the patients who were treated by “pull-through.” In general, younger women are less at risk to develop anal incontinence. The mean age in our series was 26 years. The latter technique appears seducing because it helps avoid a colostomy, which is socially and hygienically difficult to handle in Africa, among other reasons because colostomy bags are hard to purchase.

Because our treatment addressed solely the fecal incontinence, we did not focus on urinary incontinence, which was present in 80 %. Consequently, we studied the aspect of fecal incontinence to evaluate quality of life. We used the “Cleveland Clinic Incontinence Score” for this purpose rather than the Gastrointestinal Quality of Life Index (GIQLI) because the former score is confined to fecal incontinence, is easier to translate in Kiswahili and is easier to understand overall by poorly educated people (90 % of our patients were farmers).

This minimally invasive technique has allowed us to treat high rectovaginal fistulas, including the large ones as well as the ones accompanied by type II fibrosis. It permitted to avoid performing a derivative stoma, while keeping blood loss and operative morbidity minimal. Clinical success was achieved in 90 % of the 10 cases with a median follow-up of 14.3 months.
